# Effects of B-azolemiteacrylic on life-history traits and demographic parameters of two-spotted spider mite, *Tetranychus urticae* (Acari: Tetranychidae)

**DOI:** 10.1007/s10493-021-00678-4

**Published:** 2021-11-16

**Authors:** Suqin Shang, Yun Chang, Wei-Zhen Li, Wang Chang-Qing, Nie Peng-Cheng

**Affiliations:** College of Plant Protection, Biocontrol Engineering Laboratory of Crop Diseases and Pests of Gansu Province, Gansu Agricultural University, Lanzhou, 730070 China

**Keywords:** *Tetranychus urticae*, B-azolemiteacrylic, Toxicity, Sublethal effects, Life table

## Abstract

The present study was conducted to evaluate sublethal effects of B-azolemiteacrylic on the two-spotted spider mite, *Tetranychus urticae* Koch (Acari: Tetranychidae). Female adults of *T. urticae* were exposed to LC_10_ and LC_30_ of the acaricide, and the effects on treated females and their offspring were evaluated. The results showed that the fecundity of F_0_ female adults treated with LC_10_ and LC_30_ of B-azolemiteacrylic was reduced by 30.9 and 39.2%, respectively. Longevity and oviposition period of the females were significantly reduced as well. The developmental duration of egg and deutonymph stage of the F_1_ generation were not significantly different from that of the control. The protonymph stage after LC_30_ treatment lasted significantly longer, whereas the larva, deutonymph and female stage were significantly shorter than the control. The oviposition period of the F_1_ generation was significantly shortened, the fecundity of each female decreased significantly, and the ratio of female-to-male was reduced too. Moreover, the average generation period of *T. urticae* after LC_10_ and LC_30_ treatments was shorter than that of the control, and the net production rate (*R*_0_), intrinsic rate of increase (*r*_*m*_) and finite rate of increase (*λ*) were all reduced by 33.3, 7.5 and 1.9% (LC_10_ treatment) and by 51.3, 14.8 and 3.6% (LC_30_ treatment), respectively. The population doubling time was prolonged by 7.5 and 14.8% after LC_10_ and LC_30_ treatments, respectively, compared with the control. These results indicate that B-azolemiteacrylic may effectively inhibit the development rate of the F_0_ and F_1_ populations of *T. urticae*, which will help design integrated strategies for the comprehensive control of *T. urticae* and rational use of pesticides in the field.

## Introduction

The two-spotted spider mite, *Tetranychus urticae* Koch (Acari: Tetranychidae), is a destructive pest that causes serious damage to a wide range of crops and its host plants are over 140 families and 1100 species, including field crops, vegetables, fruits, and ornamental plants such as cotton, peach, strawberry, cucumber, soybean, eggplant, etc. (Maleknia et al. 2016; Mollaloo et al. 2017; Najafabadi et al. 2019). *Tetranychus urticae* uses its mouthparts to penetrate host cells and ingest cell contents (Wang et al. 2016), causing the leaves to lose green quickly until they wither and fall off. The population of *T. urticae* can be easily expanded because of its short life cycle and high reproductive potential (Saito et al. 2010; Nauen et al. 2001).

Using acaricides is the most common method to control *T. urticae* in recent years. However, the wide application of acaricides not only enables *T. urticae* to develop resistance (Brattsten et al. 1986; Van Leeuwen et al. 2010) but also leads to side effects on humans (García-Marí and González-Zamora 1999) and non-target organisms (Croft 1990), as well as the outbreak of secondary pests (Elzen 2001). One of the most used methods to manage resistance development and the conservation of biological agents is reduction of applied concentration (He et al. 2013; Song et al. 2013). Sublethal effects can be very delicate and affect populations at lower concentrations than the traditional ones (Stark and Banks 2003). In some cases, sublethal effects of pesticides can be integrated into pest control (Wang et al.^2016^). For instance, sublethal concentrations may increase pest developmental duration and reduce adult fecundity and longevity (Wang et al. 2016; Elzen 2001). Sublethal concentrations have also been applied to assess the selectivity of pesticides to beneficial mites (Alinejad et al. 2016, 2020; Bozhgani et al. 2019; Shahbaz et al. 2019). So, it is important to understand the sublethal effects and risks of acaracide application.

B-azolemiteacrylic shows excellent inhibition effects on mitochondrial respiratory chain complexes II, which mainly kills mites through contact and gastric toxicity. It also has quick effect, long duration of efficacy, broad spectrum of pests and low toxicity to non-target organisms such as bees, silkworms, fish and birds, and no interactive resistance to conventional acaricides such as abamectin and cypermethrin. It is safe for crops and environmentally friendly, and can meet the needs of integrated pest control (Song et al. 2017; Gong et al. 2017; Li 2016).

After application in the field, its toxicity will gradually decrease to sublethal doses with the extension of time and the change of environment. In addition to directly killing the target mites, some individuals will survive due to uneven application of the acaricide and other reasons, and suffer sublethal effects. As a result, the structure and size of the mite population will change again, and secondary pests will probably rise to become the primary ones (Quan et al. 2016; Han 2011). Therefore, understanding the sublethal effects of acaricides is key to evaluating their efficacy and acaricide risk management. Besides, there have been no reports on the sublethal effect of B-azolemiteacrylic on *T. urticae*. In the present study, the LC_10_ and LC_30_ of B-azolemiteacrylic were applied to *T. urticae* to investigate sublethal effects using the life-table method, and the related parameters were analyzed, aiming to evaluate the influence of sublethal effects on the development and reproduction of *T. urticae*, and to provide practical information for the rational use of B-azolemiteacrylic and comprehensive control of *T. urticae* in the field.

## Material and methods

### Mite colony maintenance and host plant

The stock population of *T. urticae* was originally obtained from Xinglong Mountain, Gansu Province, China, in May 2012, and it is known as a susceptible strain. Mites were reared on bean leaves (*Phaseolus vulgaris* L.) under acaricide-free conditions in an incubator at 25 ± 1 °C,75 ± 5% RH, and L16:D8 photoperiod.

### Acaracide preparation

In this research a commercial formulation of B-azolemiteacrylic was used (SYP-9625, suspension concentrate 30%), produced by Baozhuo, Sinochem Crop Protection Products, China.

### Concentration–response bioassay

Toxicity of pesticides to adults of two-spotted mites was tested using the leaf-dipping method (Meng 2002). A bean leaf was placed on a wet sponge in a Petri dish (7 cm diameter) and was surrounded with wet cotton to prevent the escape of mites. Thirty female adult spider mites were transferred to the leaf and prepared for bioassay. The control bean leaf was dipped with distilled water. B-azolemiteacrylic was diluted with distilled water, and five concentrations were prepared for testing: 0.8, 0.4, 0.2, 0.1 and 0.05 mg L^−1^. Every bean leaf with 30 adult spider mite females as mentioned above was dipped into each of the five B-azolemiteacrylic solution for 5 s, and then they were put in Petri dishes after blotting the spare pesticides. Concentration–response bioassay, comprising five concentrations and control, was carried out in four replications, with 180 females per replication and total sample size 720 females. The mortality covered the range of 10–90%. The LC_50_ value has a 95% confidence limit.

The mortality of adult females was recorded after 48 h of applying B-azolemiteacrylic. Mites were considered as dead if they did not show any reaction when touched by a brush. The Petri dishes were stored in a cabinet at 25 ± 1 °C, 75 ± 5% RH, and L16:D8 photoperiod.

### Assessment of sublethal effects on F_0_ and F_1_ generations

Pre-ovipositional adult females from the stock population were transferred to fresh bean leaf discs (20 mites per 7-cm-diameter disc), each of which placed on wet cotton on a sponge in a Petri dish. After about 30–60 min, the discs were dipped for 5 s in distilled water (control) or B-azolemiteacrylic at LC_10_ or LC_30_. The sample size was 600 females. After 48 h, each survived female mite (F_0_ generation) was carefully moved to a new, fresh bean leaf disc with one adult male, which ensure that the pair could mate. Each concentration included 60 pairs. The females’ longevity and fecundity were recorded every 12 h until death. Eggs (F_1_ generation) laid by F_0_ generation were collected and transferred to new leaf discs, and each leaf disc only contained one egg. Each concentration included 60 eggs. Hatching rate and development of F_1_ generation were observed every 12 h. After they entered the adult stage, the sex ratio of F_1_ was calculated. Then all the females were subjected to further rearing, each paired with one male in a disc for 1 day. The longevity and fecundity were monitored until all females died.

### Statistical analysis

In order to determine the LC values and sublethal concentrations, we used IBM SPSS v.24.0. The data obtained from F_1_ *T. urticae* were analyzed by one-way ANOVA followed by Tukey's honestly significant difference (HSD) test. Development duration, longevity, fecundity and demographic parameters of F_1_ *T. urticae* individuals were analyzed according to the two-sex life table procedure by using the Bootstrap method with 100,000 resamplings (Chi and Liu 1985; Chi 1988; Huang and Chi 2012). The paired bootstrap test was used to compare differences (Chi 2018). The computer program TWOSEX-MSChart (Chi 2018) was used to analyze the raw data. The survival rate curve was constructed using Kaplan–Meier test in IBM SPSS v.24.0.

## Results

### Estimation of LC_10_ and LC_30_ of B-azolemiteacrylic to ***Tetranychus urticae***

The LC_50_ values of B-azolemiteacrylic on *T. urticae* was estimated to be 0.127 mg L^−1^ based on the leaf-dipping method, and then sublethal concentrations (LC_10_ and LC_30_) were calculated to be 0.043 and 0.009 mg L^−1^, respectively (Table 1).

**Table 1:** Regression equation of B-azolemiteacrylic treatment for 48 h on *Tetranychus urticae*

LC_50_ regression equation	χ^2^	*R*^2^	LC_50_ (mg L^−1^) (95% CI)	LC_30_ (mg L^−1^) (95% CI)	LC_10_ (mg L^−1^) (95% CI)
y = 0.99 + 1.11x	0.210	0.991	0.127 (0.089–0.167)	0.043 (0.022–0.065)	0.009 (0.002–0.019)

### Sublethal effects of B-azolemiteacrylic on F_0_ generation

**Fig. 1:**
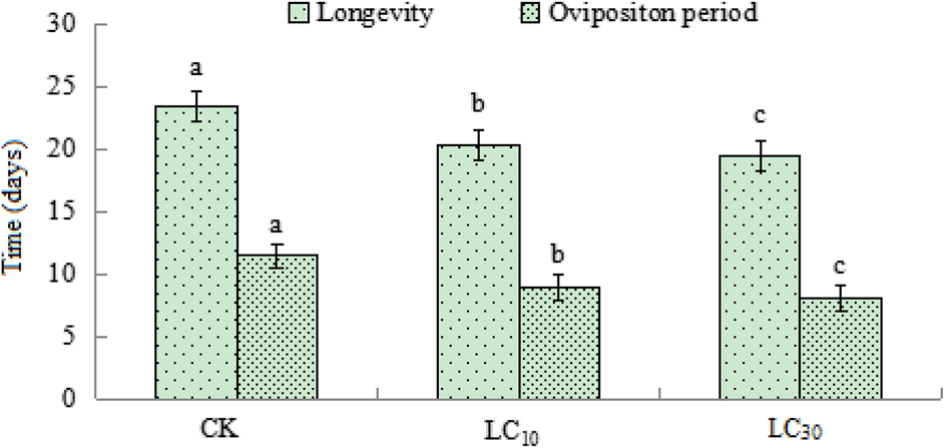
Mean (± SE) longevity and oviposition period (days) of *Tetranychus urticae* treated with sublethal concentrations of B-azolemiteacrylic. Means capped with a different letter are significantly different (Tukey’s HSD test: Ρ < 0.05)

After being treated with B-azolemiteacrylic at sublethal doses LC_10_ and LC_30_ for 48 h, the influence on their longevity and oviposition period was recorded. Longevity and oviposition period of adult females were significantly shortened after being treated with LC_10_ and LC_30_ of B-azolemiteacrylic (Fig. 1). Compared to the control’s 23.4 days, the longevity was reduced by 13.4% (LC_10_) and 17.1% (LC_30_); the oviposition period dropped from 11.46 days (control) to 8.95 days (LC_10_) and 8.05 days (LC_30_). Besides, the longevity and oviposition period at LC_30_ treatment were significantly shorter than at LC_10_ (Fig. 1).

The total and daily fecundity of the treated mites were significantly lower than of the control (Table 2). Total fecundity for untreated mites was 76.1 eggs/individual, whereas this was reduced by 30.9% (LC_10_) and 39.2% (LC_30_) after treatment. Compared with the control, the daily fecundity of each female dropped by 11.7% (LC_10_) and 16.6% (LC_30_). Total and daily fecundity at LC_30_ treatment were significantly lower than at LC_10_ (Table 2). The hatching rate (χ^2^ = 1.604, d.f. = 2, *P* = 0.45) and sex ratio (χ^2^ = 1.343, d.f. = 2, *P* = 0.51) of the F_1_ generation did not differ among the three treatments.

**Table 2:** Effects of treatment with two sublethal concentrations of B-azolemiteacrylic on mean (± SE) fecundity parameters of F_0_ generation of *Tetranychus urticae*

Treatment	n	Total fecundity (no. eggs/female)	Daily fecundity (no. eggs/female/day)	F_1_ hatching rate (%)	F_1_ sex ratio (% daughters)
Control	57	76.13 ± 1.02a	6.67 ± 0.25a	98.18 ± 0.004a	83.10 ± 0.007a
LC_10_	54	52.58 ± 0.77b	5.89 ± 0.14b	93.29 ± 0.011a	77.58 ± 0.009a
LC_30_	53	46.26 ± 1.64c	5.65 ± 0.13b	92.08 ± 0.006a	74.83 ± 0.016a

Means within a column followed by different letters are significantly different (Tukey’s HSD test: P < 0.05)

### Sublethal effects of B-azolemiteacrylic on F1 generation

The larva and adult periods and the average female longevity of the treated mites were significantly shortened (Table 3); at the LC_10_ treatment they were decreased by 5.4,13.3 and 8.0% respectively, whereas at the LC_30_ treatment reduction was 11.3, 17.4 and 9.4%, respectively. There were no significant differences in duration of the egg and deutonymph stages among all the treatments (Table 3).

**Table 3:** Effects of treatment with two sublethal concentrations of B-azolemiteacrylic on mean (± SE) developmental duration (days) of F_1_ generation of *Tetranychus urticae*

Treatment	Egg (days)	Larva (days)	Protonymph (days)	Deutonymph (days)	Adult (days)	Female longevity (days)
Control	4.49 ± 0.04a	2.03 ± 0.01a	1.71 ± 0.01b	2.16 ± 0.03a	13.94 ± 0.30a	24.51 ± 0.36a
LC_10_	4.53 ± 0.07a	1.92 ± 0.02bc	1.85 ± 0.04ab	2.15 ± 0.04a	12.09 ± 0.49b	22.56 ± 0.63b
LC_30_	4.55 ± 0.06a	1.80 ± 0.03c	1.91 ± 0.09a	2.15 ± 0.05a	11.52 ± 0.53b	22.20 ± 0.43b

Means within a column followed by different letters are significantly different (Tukey’s HSD test: P < 0.05)

The pre-oviposition period of the F_1_ generation in treatment had no significant difference from that of control, whereas the oviposition period was reduced by 20.1 and 20.7% at LC_10_ and LC_30_, respectively (Table 4). The post-oviposition period was significantly prolonged relative to the control, by 6.5% (LC_10_) and 10.6% (LC_30_). The total fecundity after LC_10_ and LC_30_ treatment was significantly lower than that of the control; it was decreased by 11 and 20.2%, respectively. Compared to the control, the sex ratio of F_2_ generation was also decreased (Table 4).

**Table 4:** Effects of treatment with two sublethal concentrations of B-azolemiteacrylic on mean (± SE) developmental duration (days) and fecundity parameters of F_1_ generation *Tetranychus urticae*

Treatment	n	Pre-oviposition (days)	Oviposition (day)	Post-oviposition (day)	Total fecundity (eggs/female)	Sex ratio (% daughters)
Control	56	1.43 ± 0.02a	12.15 ± 0.27a	1.44 ± 0.03b	70.21 ± 1.30a	81.43 ± 0.014a
LC_10_	47	1.39 ± 0.03a	9.71 ± 0.37b	1.54 ± 0.01a	62.49 ± 2.30b	77.59 ± 0.005a
LC_30_	43	1.38 ± 0.02a	9.64 ± 0.31b	1.61 ± 0.02a	56.06 ± 1.69c	75.60 ± 0.011a

Means within a column followed by different letters are significantly different (Tukey’s HSD test: P < 0.05)

**Fig. 2:**
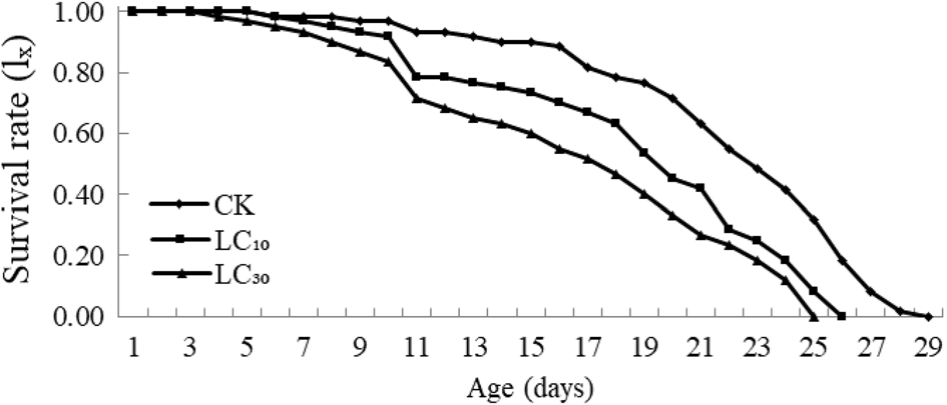
Age-specific survival rate (Ix) for F_1_ generation of *Tetranychus urticae* treated with sublethal concentrations of B-azolemiteacrylic

The survival curves of F_1_ generation were similar with that of the control (χ^2^ = 1.627, d.f. = 2, *P* = 0.44), all of type I (arched curve) (Fig. 2). The survival rate of both treatments were lower than that of the control except for the egg stage, and treated mites lived shorter than mites of the control group.

**Fig. 3:**
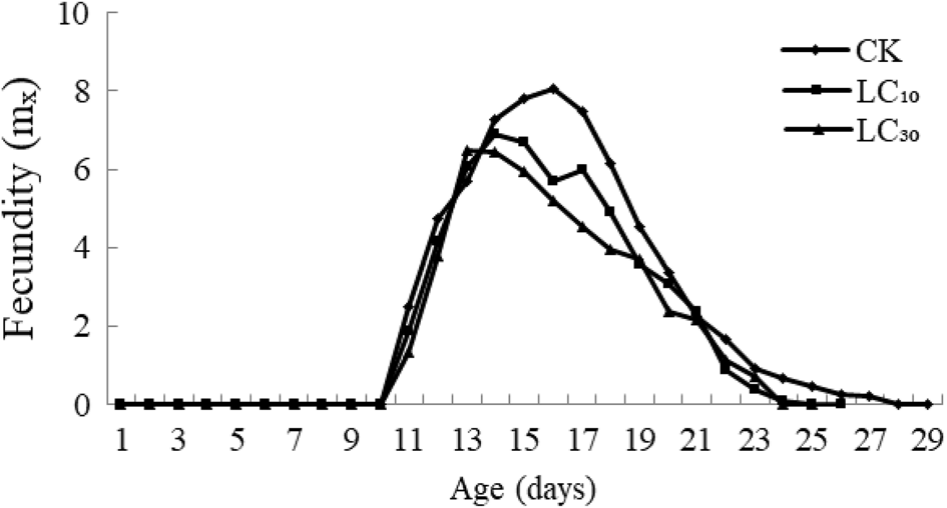
Age-specific fecundity (m_x_) for F_1_ generation of *Tetranychus urticae* treated with sublethal dosage of B-azolemiteacrylic

Fecundity (Mx, the average number of females produced by a female mite) earliest at LC_30_ treatment (on day 13), then at LC_10_ (day 14) and latest at the control (day 16). The peak was highest for the control, and lowest for the LC_30_ treated mites (Fig. 3), indicating that the capability of each adult to produce females decreased after being with a sublethal dose of B-azolemiteacrylic.

The net reproductive rate (*R*_0_) of both treatments was significantly lower than that of the control group—compared to the control, *R*_*0*_ was 33.3% (LC_10_) and 51.3% (LC_30_) lower (Table 5), indicating that the B-azolemiteacrylic had a great impact on the fecundity of the F_1_ generation. Compared with the control group, the mean generation time (*T*), the intrinsic rate of increase (*r*_*m*_), the finite rate of increase (λ), and the population doubling time for mites treated with both sublethal concentrations of B-azolemiteacrylic did not differ significantly (Table 5).

**Table 5:** Effects of treatment with two sublethal concentrations of B-azolemiteacrylic on mean (± SE) biological parameters of F_1_ generation *Tetranychus urticae*

Treatment	Net reproductive rate (*R*_*0*_) (no. offspring/individual)	Mean generation time (*T*) (days)	Intrinsic rate of increase (*r*_*m*_) (day^−1^)	Finite rate of increase (*λ*) (day^−1^)	Population doubling time (days)
CK	52.74 ± 3.06a	15.82 ± 0.14a	0.25 ± 0.001a	1.28 ± 0.004a	2.77 ± 0.07a
LC_10_	35.20 ± 1.65b	15.40 ± 0.08a	0.23 ± 0.004a	1.26 ± 0.003a	2.99 ± 0.03a
LC_30_	25.69 ± 1.98c	15.20 ± 0.21a	0.21 ± 0.003a	1.24 ± 0.001a	3.25 ± 0.05a

Means within a column followed by different letters are significantly different (Tukey’s HSD test: P < 0.05)

## Discussion

In the present study, the biological parameters and demographic data related to different generations of *T. urticae* were investigated by applying sublethal concentrations of B-azolemiteacrylic. In recent years, a number of studies have been conducted for evaluating the lethal and sublethal effects of various pesticide groups such as tetrazine, tetronic acid, pyrazolium, pyrethroid, organophosphate, pyridine azomethines, and neonicotinoid derivatives on two-spotted spider mites, as well as its predatory mites (Hamedi et al. 2010, 2011; Lima et al. 2013; Alinejad et al. 2016; Bozhgani et al. 2018; Havasi et al. 2021). As one of the effective acrylonitrile group acaricides, however, no sublethal effects of B-azolemiteacrylic on biological parameters of *T. urticae* were known.

Our study indicated that when treated by B-azolemiteacrylic at LC_30_, the protonymph stage was significantly prolonged, and the larvae stage, adult stage and average life span were shortened. In addition, the oviposition period, fecundity and sex ratio from mites of the F_1_ generation treated at LC_10_ and LC_30_ were also decreased. These results corresponded with those of Havasi et al. (2018), in which the experimental concentration of diflovidazin played a negative role during all pre-adult developmental stages such as the egg, larva, protonymph, and deutonymph among males. Regarding females, no significant difference was observed between the immature stages for all the tested concentrations, except in egg and protonymph stages. Similar results were also seen in other investigations (Fan 2015; Tian 2017; Gao 2018). On the contrary, an increase in the concentration caused a significant difference during immature stages of *T. urticae* in males and females when treated by sublethal concentrations of bifenazate (Li et al. 2017). This might be caused by a different working mechanism of the two agents.

The results of the present study indicated the sublethal concentration had a certain inhibitory influence on the population growth of F_0_ generation, which was specifically displayed in decreases of longevity, oviposition period, fecundity and hatching rate, sex ratio of the next generation; the higher the concentration, the greater the degree in reduction. Negative sublethal effects of a variety of acaricides on, for instance, fecundity, life span, and oviposition period of pest mites have been reported by many researchers (Yong et al. 2011; Tao and Wu 2006; Xin et al. 2019; Li et al. 2016; Bozhgani et al. 2019; Havasi et al. 2020). Our results were consistent with those of Alinejad et al. (2015), in which a significant decrease happened in longevity after being treated with sublethal concentrations of fenazaquin. Similarly, a significant decrease occurred in the longevity for mites treated with azadirachtin at 64 and 128 ppm (Martínez-Villar et al. 2005), the reduction in fecundity was shown after treatment with a sublethal dose of spiromesifen (Marcic 2005). Reduction of the oviposition period can decrease the next-generation population size. Shortening of the life span would not only restrain fecundity, but also lower the potential damage caused by pest mites to their hosts.

Life-table parameters play a vital role in the comprehensive evaluation of the controlling effect of pesticides against mites. It is recommended to evaluate the sublethal effect of agents on target pests with the instantaneous rate of increase (*r*) or intrinsic rate of increase (*r*_*m*_) of the population, and conduct a comprehensive study with the life table technology (Stark and Wennergren 1995, Stark and Banks 2003). In this study, the net reproductive rate (*R*_0_) following the treatment of females from F_0_ generation with sublethal concentrations of B-azolemiteacrylic was significantly lower than that of the control group, but the intrinsic rate of increase (*r*_*m*_) and finite rate of increase (*λ*) were not significantly different from the control. The results were congruent with those of Wang et al. (2014a, b) and Marcic (2007), in which the sublethal doses of bifenthrin (LC_10_ and LC_25_) and spirodiclofen (6, 12, 24, 48, and 96 mg L^−1^) were examined on the two-spotted spider mite, respectively. Similar results about the effect of triflumuron on *T. urticae* were also seen in the study of Sáenz-de-Cabezón et al. (2006).

Based on the results of the present study, the exposure to sublethal concentrations of B-azolemiteacrylic had a negative effect on biological parameters of *T. urticae* (i.e., lower *R*_*0*_). B-azolemiteacrylic sublethal doses could effectively inhibit the developmental rates of F_0_ and F_1_ populations of *T. urticae*, and the higher the concentration, the stronger the inhibition effect. Besides, no proliferation effect was found in *T. urticae* population, which suggests that *T. urticae* may not easily develop resistance to B-azolemiteacrylic. This advantage is of positive significance to the formulation of integrated management strategies for *T. urticae*. Consequently, it is recommended that applying B-azolemiteacrylic at lower rates could lead to effective control of *T. urticae*. Nevertheless, most of the similar experiments including ours carried out under laboratory conditions may not be fully representative of a natural field, because environmental complexity, different plants and other natural characteristics cannot be 100% replicated in a small room. Further experiments carried out under greenhouse and field conditions are therefore needed.
